# Choline ameliorates cardiovascular damage by improving vagal activity and inhibiting the inflammatory response in spontaneously hypertensive rats

**DOI:** 10.1038/srep42553

**Published:** 2017-02-22

**Authors:** Longzhu Liu, Yi Lu, Xueyuan Bi, Man Xu, Xiaojiang Yu, Runqing Xue, Xi He, Weijin Zang

**Affiliations:** 1 Department of Pharmacology, School of Basic Medical Sciences, Xi’an Jiaotong University Health Science Center, Xi’an, Shaanxi, 710061, People’s Republic of China

## Abstract

Autonomic dysfunction and abnormal immunity lead to systemic inflammatory responses, which result in cardiovascular damage in hypertension. The aim of this report was to investigate the effects of choline on cardiovascular damage in hypertension. Eight-week-old male spontaneously hypertensive rats (SHRs) and Wistar-Kyoto rats were intraperitoneally injected with choline or vehicle (8 mg/kg/day). After 8 weeks, choline restored the cardiac function of the SHRs, as evidenced by decreased heart rate, systolic blood pressure, left ventricle systolic pressure, and ±dp/dt_max_ and increased ejection fraction and fractional shortening. Choline also ameliorated the cardiac hypertrophy of the SHRs, as indicated by reduced left ventricle internal dimensions and decreased cardiomyocyte cross-sectional area. Moreover, choline improved mesenteric arterial function and preserved endothelial ultrastructure in the SHRs. Notably, the protective effect of choline may be due to its anti-inflammatory effect. Choline downregulated expression of interleukin (IL)-6 and tumour necrosis factor-α and upregulated IL-10 in the mesenteric arteries of SHRs, possibly because of the inhibition of Toll-like receptor 4. Furthermore, choline restored baroreflex sensitivity and serum acetylcholine level in SHRs, thus indicating that choline improved vagal activity. This study suggests that choline elicits cardiovascular protective effects and may be useful as a potential adjunct therapeutic approach for hypertension.

Hypertension is a major cardiovascular risk factor that affects approximately one-third of the world’s population^1^. It may lead to various types of cardiovascular damage, such as cardiac remodelling^2^, renal dysfunction^3^, stroke^4^ and arterial stiffening^5^. Despite significant progress in the diagnosis and treatment of hypertension, the pathophysiology of hypertension is complex and generally poorly understood. Recent studies have suggested that the immune system may play a critical role in hypertension by participating in inflammatory responses in the central nervous^6^, renal^7^ and cardiovascular systems^8^. In addition, a growing body of evidence suggests that systemic inflammation leads to cardiovascular damage and cardiac hypertrophy in patients with hypertension^9^,^10^,^11^,^12^. Toll-like receptor 4 (TLR4), an important component of the innate immune system that is expressed on the surface of several cell types, including endothelial and vascular smooth muscle cells, plays an important role in mediating the inflammatory response in hypertension^8^,^13^. Recently, Bomfim *et al*. have demonstrated that TLR4 protein expression in mesenteric arteries is higher in spontaneously hypertensive rats (SHRs) compared with Wistar-Kyoto (WKY) rats and that inhibiting TLR4 activation by treating these rats with an anti-TLR4 antibody results in decreased blood pressure and IL-6 levels in the serum as well as reduced vascular hypercontractility^14^. Therefore, the inflammatory response has emerged as an attractive therapeutic target for the treatment of hypertension.

It is well known that the activation of efferent vagal nerve fibres can modulate local and systemic inflammatory responses, known as the ‘cholinergic anti-inflammatory pathway’. However, the anti-inflammatory activity of the vagal nerve is reduced and the pro-inflammatory activity of sympathetic nerve is increased in hypertension^15^. In addition, it has been reported that SHRs show deficits in the cholinergic anti-inflammatory pathway^16^,^17^, and these deficits appear to contribute to the pathogenesis of end-organ damage in hypertension. Recent evidence has implicated dysfunctional neural-immune regulation in the pathogenesis of hypertension^18^,^19^. Conventional hypertension therapies focus on strategies for attenuating sympathetic nerve activity, whereas the possibility of improving vagal nerve activity has generally been neglected. A recent study showed that chronic vagal nerve stimulation alleviates hypertension-induced endothelial dysfunction and aortic stiffening in stroke-prone SHRs^20^. Therefore, increasing vagal activity may be an interesting alternative approach for antihypertensive therapy, and it is therefore necessary to find effective pharmaceutical therapies for the improvement of vagal activity in hypertension.

Choline, a safe and effective medicine, has been used in the clinical treatment of steatohepatitis. As a precursor of acetylcholine, choline also has protective effects against various cardiovascular diseases such as myocardial infarction^21^, arrhythmias^22^, cardiac hypertrophy^23^,^24^ and ischaemia/reperfusion injury^25^. Our recent studies have shown that choline exhibits a remarkable protective effect against ischaemia/reperfusion-induced vascular damage in rats by inhibiting the reactive oxygen species-mediated Ca^2+^/calmodulin-dependent protein kinase II pathway and regulating Ca^2+^-cycling proteins^26^. However, the effects of choline on the inflammatory response and vagal activity, two important factors in hypertension, have not been characterized in SHRs. Therefore, in the present study, we sought to investigate the effects of choline on vagal activity in hypertension, as proposed in a recent presentation by the authors^27^. Additionally, the role of choline in inhibiting the inflammatory response and ameliorating cardiovascular damage in SHRs is also explored here.

## Results

### Choline attenuated the development of hypertension, improved cardiac function, and increased baroreflex sensitivity and serum ACh level in SHRs

**Figure 1:**
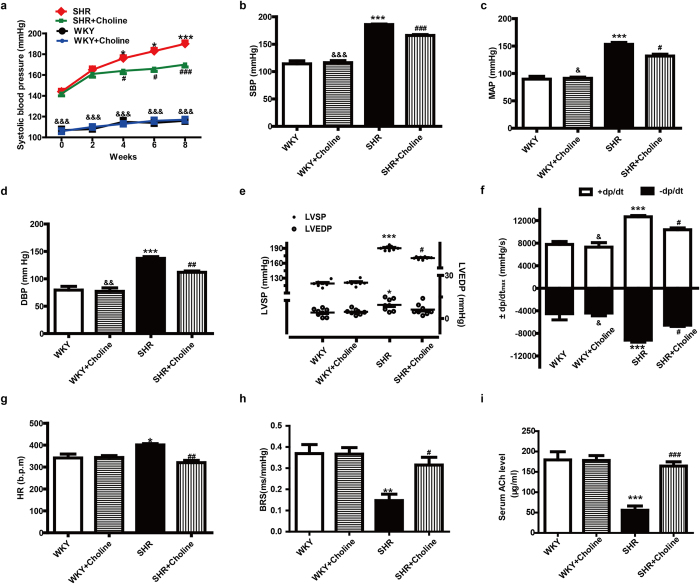
Choline attenuated the development of hypertension, improved cardiac function, and increased BRS and serum ACh level in SHRs. (**a**) SBP for eight weeks measured by tail cuff. (**b**) SBP, (**c**) MAP, (**d**) DBP, (**e**) LVSP & LVEDP, (**f**) ±dp/dt_max_ and (**g**) HR were measured by polygraph recorder at the end of the experiment. (**h**) The vagal activity index, BRS. (**i**) The concentration of ACh level in serum. Data are mean ± SEM (n = 8). ^*^P < 0.05, ^**^P < 0.01, ^***^P < 0.001 vs WKY. ^#^*P* < 0.05, ^##^*P* < 0.01, ^###^*P* < 0.001 vs SHR. ^&^*P* < 0.05, ^&&^*P* < 0.01, ^&&&^*P* < 0.001 vs SHR+Choline.

The systolic blood pressure (SBP) of the SHR group was significantly higher than that of the WKY group and the WKY+Choline group throughout the course of the experiment. After eight weeks of choline therapy, the SBP of the SHR+Choline group, as measured by tail cuff in conscious rats, was significantly lowered to 170 ± 3.0 mmHg compared with 190 ± 4 mmHg in SHRs, though it was still higher than that of the WKY+Choline group (117 ± 2.0 mmHg). There were no marked differences in the SBP of the WKY group (116 ± 1.0 mmHg) compared with the WKY+Choline group (117 ± 2.0 mmHg) (Fig. 1a). These data suggested that choline attenuated the development of hypertension. After the 8-week choline treatment, the haemodynamic parameters of the anaesthetized 16-week-old rats were measured and analysed by a polygraph recorder. The SBP, mean artery pressure (MAP), diastolic blood pressure (DBP), left ventricle systolic pressure (LVSP), maximum rate of rise/descent of left ventricular pressure (±dp/dt_max_) and heart rate (HR) in the SHR+Choline group, compared with the SHR group, were markedly decreased (Fig. 1b–g). There were no significant differences in SBP, MAP DBP, LVSP, ±dp/dt_max_ or HR between the WKY+Choline and WKY groups. Baroreflex sensitivity (BRS), a marker of vagal activity, was decreased in the SHR group compared with the WKY group and was restored by choline treatment in the SHR+Choline group (Fig. 1h). We next attempted to clarify the effect of choline on serum ACh levels. As depicted in Fig. 1i, the concentration of ACh in serum decreased significantly in the SHR group compared with the WKY group. The choline treatment notably increased the concentration of ACh in the SHR+Choline group. No significant differences in serum ACh levels were found among the WKY, WKY+Choline and SHR+Choline groups.

### Choline attenuated cardiac hypertrophy in SHRs

**Figure 2:**
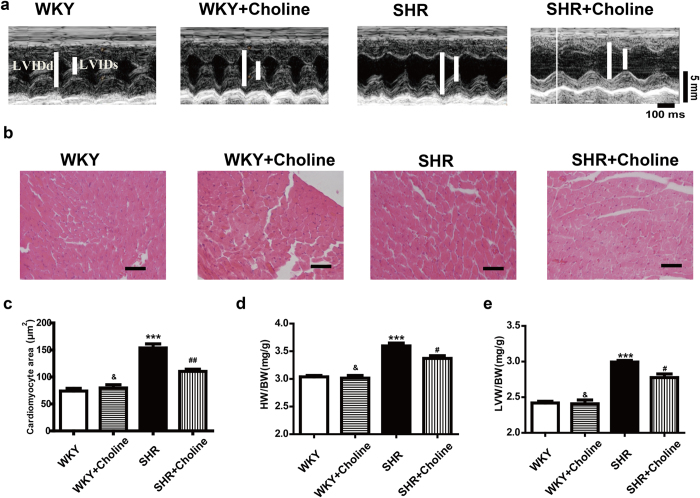
Choline attenuated cardiac hypertrophy in SHRs. (**a**) Representative H&E staining of cardiac cross-section. (**b**) Quantification of cross-sectional areas of the cardiomyocytes. (**c**) Quantification of cross-sectional areas of the cardiomyocytes. (**d**) The ratio of HW/BW. (**e**) The ratio of LVW/BW, Scale bar = 30 μm. Data are mean ± SEM (n = 8). ^*^P < 0.05, ^**^P < 0.01, ^***^P < 0.001 vs WKY; ^#^*P* < 0.05, ^##^*P* < 0.01 vs SHR; ^&^*P* < 0.01 vs SHR+Choline.

Compared with the WKY group, the SHR group exhibited increased left ventricle internal dimension in systole and diastole (LVIDs and LVIDd), thickness of the left ventricle posterior wall (LVPW), end-systolic volume (ESV), end-diastolic volume (EDV), and cardiac output (CO) and decreased left ventricle ejection fraction (LVEF) and left ventricle ejection fractional shortening (LVFS). (Table 1, Fig. 2a). Eight weeks of treatment with choline ameliorated these pathological changes in the SHR+Choline group compared with the SHR group. A similar result was found in comparing the cross-sectional area of cardiomyocytes, observed by H&E staining, among different groups. The SHR+Choline group showed an obvious decrease in the cross-sectional area of cardiomyocytes (Fig. 2b,c). The ratios of HW/BW (Fig. 2d) and LVW/BW (Fig. 2e) also revealed that choline attenuated the cardiac hypertrophy of SHRs. No differences were observed between the WKY group and the WKY+Choline group. These data suggested that choline significantly attenuated the cardiac hypertrophy of SHRs. Compared with the WKY group, the SHR group exhibited decreased LVEF and LVFS and increased LVSP and ±dp/dt_max_. Eight weeks of treatment with choline reversed the pathological changes and improved the cardiac function of SHRs. No differences were observed between the WKY group and the WKY+Choline group.

### Effects of choline on renal damage in SHRs

**Figure 3:**
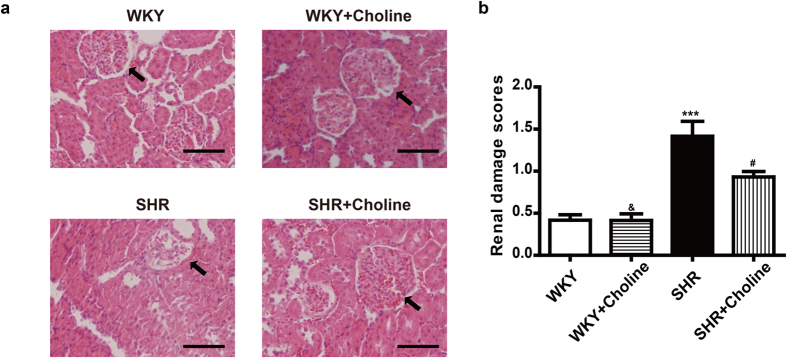
Choline attenuated renal damage in SHRs (**a**) Representative renal section with H&E staining in the experimental groups, arrows indicate glomeruli, scale bar = 50 μm. (**b**) The renal damage scores. Data are mean ± SEM (n = 8). ^***^P < 0.001 vs WKY; ^#^*P* < 0.05 vs SHR; ^&^*P* < 0.01 vs SHR+Choline.

As is shown in Fig. 3, a histopathological examination showed no obvious pathological glomerular or tubular changes in the WKY group and the WKY+Choline group. However, collapsed glomeruli and the disappearance of the renal tubular brush border were observed in SHRs (Fig. 3a). Although the renal damage scores in the SHR+Choline group were significantly higher than those in the WKY+Choline group (Fig. 3b), choline markedly ameliorated renal damage in SHRs compared with the SHR group.

### Effects of choline on endothelial dysfunction and damage in SHRs

**Figure 4:**
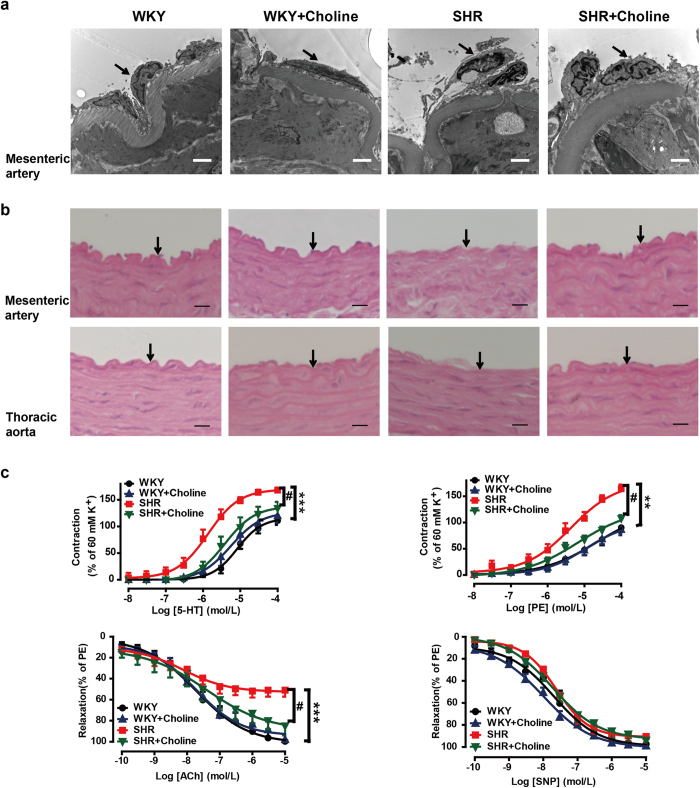
Choline ameliorated the endothelial damage in SHRs. (**a**) Representative transmission electron micrographs of mesenteric artery. Arrows indicate endothelial cells. Scale bar = 2 μm. (**b**) Light microscopy image of H&E stained mesenteric artery and thoracic aorta, Scale bar = 10 μm. Arrows indicate endothelial cells. (**c**) Effects of choline on the concentration-response curves of mesenteric artery rings to 5-HT, PE, ACh and SNP in the mesenteric artery rings of WKY group (black), WKY+Choline group (blue), SHR group (red), SHR+Choline group (green) respectively. Data are mean ± SEM (n = 8). ^**^P < 0.01, ^***^P < 0.001 vs WKY group; ^#^*P* < 0.05 vs SHR group.

To examine whether choline protected the endothelium from structural damage in SHRs, we observed the microstructure and ultrastructure of the mesenteric artery and thoracic aorta by transmission electron microscopy (Fig. 4a) and H&E staining (Fig. 4b), respectively. Endothelial damage was demonstrated by endothelial cell desquamation from the elastic membrane and elastic membrane fragmentation in the SHR group, thus suggesting that endothelial structural degradation might have contributed to endothelial dysfunction in the SHR group. In the SHR+Choline group, the endothelial damage was ameliorated. Endothelium structural damage was not found in the WKY or WKY+Choline groups.

We examined the effects of choline on the responses to 5-hydroxytryptamine (5-HT, 10^−8^–10^−4^ M), phenylephrine (PE, 10^−8^–10^−4^ M), acetylcholine (ACh, 10^−10^–10^−5^ M) and sodium nitroprusside (SNP, 10^−10^–10^−5^ M) by using endothelium-intact mesenteric artery rings (Fig. 4c). The maximum contraction induced by 5-HT in the mesenteric arteries increased in the SHR group compared with the WKY group (*E*_max_: 168% ± 5% vs 111% ± 8%; *P* < 0.001). Treatment with choline reduced the maximum contractile response to 5-HT in the SHR+Choline group (*E*_max_: 134% ± 12%, *P* < 0.05 vs the SHR group). No significant differences were found among the SHR+Choline, WKY+Choline and WKY groups (*E*_max_: 134% ± 12% vs 120% ± 5% vs 111% ± 8%, *P* > 0.05). Similarly, a significantly enhanced maximum contractile response to PE was detected in the SHR group (*E*_max_: 164% ± 8%) compared with the WKY group (*E*_max_: 90% ± 5%; *P* < 0.01). Choline treatment decreased the maximum contractile response to PE in the SHR+Choline group (108% ± 6%, *P* < 0.05 vs the SHR group). The maximum contractile response to PE was not affected by choline in the WKY+Choline group compared with the WKY group (*E*_max_: 86% ± 4% vs the WKY group, *P* > 0.05). The maximum ACh-induced vasorelaxation was significantly attenuated in mesenteric arteries from the SHR group compared with the WKY group (*E*_max_: 51% ± 6% vs 99% ± 7%, *P* < 0.001). Treatment with choline reversed the endothelial dysfunction, as evidenced by the enhanced maximum relaxation to ACh in the SHR+Choline group (*E*_max_: 85% ± 8%, *P* < 0.05 vs the SHR group). There were no differences among the SHR + Choline, WKY+Choline and WKY groups (*E*_max_: 85% ± 8% vs 98% ± 4% vs 99% ± 7%, *P* > 0.05). The relaxations to SNP were similar in all the experimental groups, and they were not affected by choline (*E*_max_: 97% ± 4% in the WKY group, 99% ± 1% in the WKY+Choline group, 92% ± 5% in the SHR group and 93% ± 4% the SHR+Choline group, respectively).

### Western blot analysis of TLR4 and inflammatory cytokines

**Figure 5:**
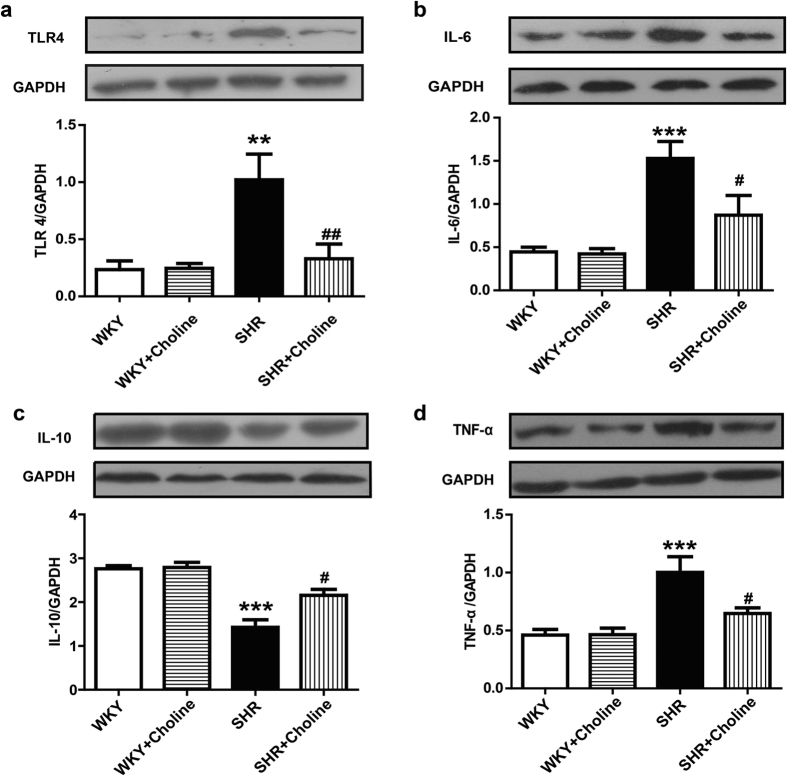
Western blot results of the expression of TLR4 protein and inflammatory cytokines in mesenteric arteries. (**a**) Protein expression of TLR4. (**b**) Protein expression of IL-6. (**c**) Protein expression of IL-10. (**d**) Protein expression of TNF-α. Data are mean ± SEM (n = 6). ^**^P < 0.01, ^***^P < 0.001 vs WKY group; ^#^*P* < 0.05, ^##^*P* < 0.01 vs SHR group. Full-length blots are presented in Supplementary Figure 5a–d.

A western blot analysis was performed to assess the role of TLR4 and inflammation in hypertension. The protein expression of TLR4, IL-6 and TNF-α in the mesenteric arteries was increased in the SHR group compared with the WKY group. Choline decreased the protein expression of TLR4, IL-6 and tumour necrosis factor-α (TNF-α) in the SHR+Choline group compared with the SHR group. The expression of IL-10, an anti-inflammatory cytokine, was decreased in the SHR group and restored in the SHR+Choline group. No differences were observed between the WKY and WKY+Choline groups (Fig. 5a–d).

## Discussion

The results of the present study provide the first evidence that choline treatment alleviates cardiovascular damage and slows the progression of hypertension possibly via the improvement of vagal activity and inhibition of the vascular inflammatory response in SHRs. The salient findings of this study are as follows: (1) SHRs displayed a series of significant characteristics, such as low vagal activity, high blood pressure, target-organ damage and cardiac hypertrophy; (2) choline treatment attenuated the development of hypertension, reduced heart rate and prevented hypertension-associated cardiac, renal and vascular damages; (3) these cardiovascular protective effects of choline were related to the inhibition of TLR4 and pro-inflammatory cytokines (IL-6, TNF-α) and the upregulation of anti-inflammatory cytokine IL-10; and (4) choline significantly increased BRS and serum ACh levels, thus indicating that choline elevated vagal activity. The anti-inflammatory effects and improvement of vagal activity induced by choline may play a role in slowing the progression of hypertension in SHRs and may provide a new possibility for antihypertensive therapy.

Left ventricular hypertrophy (LVH) is an important factor in hypertension development. The functional role of the adrenergic system in cardiac hypertrophy and heart failure is well documented. Recent studies have indicated that the amelioration of β-adrenergic receptor responsiveness through physical training contributes to clinical improvements in cardiovascular health^28^ and that intracardiac injection of AdGRK5-NT reduces LVH by inhibiting NF-kB–dependent hypertrophic gene expression^29^. In addition, the modulation of GRK and β-arrestin signalling is also a promising strategy for the treatment of cardiac remodelling in heart failure^30^. However, the role of the cholinergic system in cardiac hypertrophy in hypertension still remains to be elucidated. On the basis of the observation that choline ameliorated dysfunctional vasoconstriction and vasodilatation in the mesenteric arteries of SHRs in the present study, it is possible that the decreased peripheral resistance and subsequent afterload reduction is a mechanism by which choline ameliorated LVH. However, it has been reported that choline may directly inhibit cardiac hypertrophy by regulating transient receptor potential cation channel 6^31^. It has also been reported that choline attenuates angiotensin II-induced cardiac hypertrophy by inhibiting the p38 MAPK pathway and intracellular calcium signalling^23^. Therefore, in this study, choline may have ameliorated the LVH of SHRs in both direct and indirect ways.

Autonomic imbalance characterized by increased sympathetic activity and decreased vagal activity has been correlated with the development of hypertension^32^. Thus, it is conceivable that the increase in vagal activity may favourably affect outcome in patients with hypertension and may represent a strategic target for antihypertensive treatment. Notably, the change in blood pressure was significant only after 4 weeks of choline treatment, and there was an approximately 20 mmHg reduction in blood pressure in the SHRs at the end of the experiment; however, the cardiovascular damage in the SHRs was significantly ameliorated by choline. Several factors may have contributed to these results. Hypertension is a chronic inflammatory disease with sustained high blood pressure and cardiovascular damage^33^,^34^. The blood pressure of SHRs appears to increase when rats are approximately 6 weeks old, and the systolic blood pressure reaches approximately 180–200 mmHg^35^. We began our experiments with 8-week-old rats, and the rats were then treated with choline for 8 weeks, during the development of hypertension. The protective effect of choline may be gradual, because the development of hypertension and the choline-induced decrease in blood pressure may occur simultaneously. Furthermore, choline slowed the progression of hypertension and ameliorated cardiovascular damage. Importantly, protection against cardiovascular target-organ damage has become a part of therapeutic strategies for hypertension^4^. Moreover, there is evidence indicating that improvements in cardiac function may be independent of a decrease in blood pressure in the treatment of hypertension^36^. The acetylcholinesterase inhibitor pyridostigmine has been shown to protect the cardiovascular system in the absence of a significant reduction in blood pressure in SHRs by activating the vagal nerve and reducing inflammation^37^. It has been reported that choline attenuates angiotensin II-induced cardiac hypertrophy and improves cardiac function in mice without causing obvious changes in blood pressure^23^. Our study showed that choline treatment attenuated the development of hypertension and increased vagal activity, as demonstrated by the enhanced BRS and increased serum ACh levels. BRS is a marker of the capability of reflexes to increase vagal activity and to decrease sympathetic activity in response to a sudden increase in blood pressure^38^. It is widely used as an index of vagal activity in animal experiments^39^,^40^,^41^,^42^. In addition, ACh, the principal vagal nerve neurotransmitter, has been considered to reflect vagal activity. Direct electrical vagal stimulation has been shown to increase the myocardial interstitial ACh level^43^ and the ACh level in the bloodstream^44^. Therefore, on the basis of our results, we propose that, in addition to the reduction in blood pressure, the increase in vagal activity may partly contribute to the improvements in cardiovascular function.

A substantial amount of evidence has indicated that inflammation plays a significant role in hypertension^15^,^45^. The TLR4 signalling pathway, an inflammatory component of the innate immune response, plays an important role in hypertension^13^. Experimental evidence has indicated that the inhibition of TLR4 significantly reduces the inflammatory response and blood pressure in hypertension models^6^,^14^,^46^. In the present study, we found that TLR4 expression was increased in the mesenteric arteries from SHRs compared with WKY rats and that treatment with choline for eight weeks reduced the TLR4 expression in SHRs. IL-6, a pro-inflammatory cytokine acting downstream of TLR4, is released from many cell types, including endothelial cells^47^ and vascular smooth muscle cells^48^. Some studies have shown a close relationship between IL-6 levels and high blood pressure^46^,^49^. IL-6 knockout has been shown to attenuate angiotensin II-induced hypertension^50^. In our study, we observed a reduction in IL-6 expression in the mesenteric arteries of SHRs after choline treatment. Additionally, treatment with choline reduced the expression of TNF-α and increased the anti-inflammatory cytokine IL-10 in the vessels in SHRs. A previous study has shown that choline inhibits endotoxin-induced elevations in TNF-α^51^. Lataro *et al*. have demonstrated that donepezil reduces the plasma levels of TNF-α, IL-6, and interferon-γ, thus indicating that acetylcholinesterase inhibition attenuate the development of hypertension in SHRs, probably through anti-inflammatory effects^37^. These results may lend further support to the concept that decreases in inflammatory markers are a direct consequence of the increase in plasma choline levels. A previous study has indicated that inhibition of the inflammatory response ameliorates cardiovascular damage^52^. In the present study, our results showed that the cardiovascular damage in hypertension was ameliorated, and this was accompanied by a reduced inflammatory response, thus suggesting that the protective effect of choline may be attributed to its anti-inflammatory effects.

In the present study, the improvement of vagal activity was accompanied by decreased expression of TLR4 in SHRs. Our data do not prove that there is a relationship between the cholinergic anti-inflammatory pathway and the TLR4 signalling pathway. Nevertheless, the results from this study are promising and provide clear clues for future in depth studies. Further studies of the detailed mechanism by which choline induces the suppression of pro-inflammatory cytokines and how this process correlates with vagal activity are warranted. Moreover, renal injury plays an important role in hypertension^7^. It has been recently suggested that inflammation may alter renal function and predispose an individual to hypertension^53^. In the present study, we observed protective effects of choline against hypertension-related renal injury; these effects may have contributed to the attenuated progression of hypertension in the SHRs, although the mechanism involved requires further investigation.

Only male rats were used in our study to exclude the influence of oestrogen on the cardiovascular system. It has been suggested that oestrogen exerts a cardiovascular protective effect in hypertension^54^,^55^,^56^,^57^. Many investigations have suggested that oestrogen is involved in several mechanisms that protect against hypertension, such as the dilation of vessels by increasing nitric oxide and prostacyclin and the inhibition of vasoconstrictors through the regulation of the sympathetic nervous and angiotensin system^58^. There is evidence that the decline in oestrogen levels during menopause is a risk factor for hypertension^59^. In addition, Saleh *et al*. have suggested that females have a heightened parasympathetic tone compared with males, thus supporting a role of oestrogen as a central modulator of autonomic tone and baroreflex sensitivity^60^.

Notably, we chose to treat the rats in this study with a choline dose of 8 mg/kg/day. Recent studies have demonstrated that the administration of choline (14 mg/kg/day) to mice for 2–3 weeks results in a marked decrease in cardiac hypertrophy^23^,^24^. According to the formula for dose translation based on body surface area^61^, the dose for rats = the dose for mice × Km factor of mice/Km factor of rats = 14 × 3/6 = 7 mg/kg/day. Furthermore, an intravenous dose of 10 mg/kg choline has been used for acute administration in several previous studies of myocardial ischaemia or ischaemia/reperfusion rat models^21^,^25^. In addition, hypertension is a chronic inflammatory disease involving a sustained rise in blood pressure and cardiovascular damage and may require long-term treatment^33^,^34^. Our experimental results showed that the treatment of SHRs with 8 mg/kg/day choline for 8 weeks significantly attenuated the progression of hypertension, improved cardiovascular function and improved vagal activity.

A survey of the literature indicates that the effects of choline on either a salt-induced or drug-induced hypertension model have not been elucidated. The potential mechanism of choline treatment may be different in models of hypertension of varying aetiology. The mechanisms of salt-induced hypertension may include genetic factors, inflammation-induced kidney damage, the aldosterone–mineralocorticoid receptor and neuronal alterations^62^,^63^. Our results showed that choline inhibited the inflammatory response and attenuated kidney injury. On the basis of the data from our current study, it is possible that choline may ameliorate salt-induced hypertension through reducing inflammation-induced renal damage. Angiotensin II (Ang II) is a commonly used drug for inducing hypertension. Ang II causes increased vasoconstriction, elevated sympathetic activity, and an increased release of aldosterone and inflammatory cytokines, all of which contribute to the increase in blood pressure^64^,^65^. In this regard, the effects of choline on improving vagal activity, reducing vasoconstriction and inhibiting inflammatory cytokines indicate that choline may have a beneficial effect in treating Ang II-induced hypertension.

**Figure 6:**
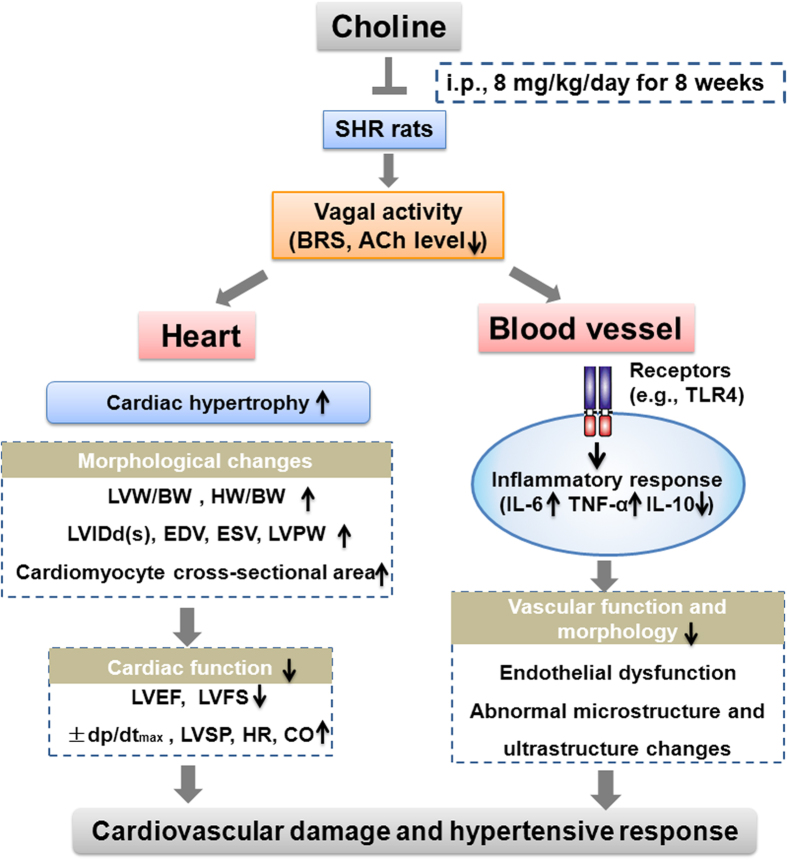
The putative schematic illustration of the mechanism underlying choline-elicited cardiovascular protection in hypertension. SHRs displayed a series of significant characteristics, including low vagal activity, high blood pressure, cardiovascular damage and cardiac hypertrophy. Choline reduced cardiac hypertrophy by decreasing the LVM/BW, HW/BW, LVIDd(s), EDV, ESV, LVPW and cardiomyocyte cross-sectional area in SHRs. Choline restored cardiac function by decreasing ±dp/dt_max_, LVSP, HR, and CO and increasing LVEF and LVFS in SHRs. Moreover, choline prevented vascular dysfunction and abnormal structure changes, possibly by reducing expression of TLR4, IL-6 and TNF-α and upregulating IL-10 in SHRs. These cardiovascular protective effects of choline may be attributed to the improvement of vagal activity and the inhibition of inflammatory responses. ACh, acetylcholine; BRS, baroreflex sensitivity; BW, body weight; CO, cardiac output; EDV, end diastolic volume; ESV, end-systolic volume; LV, left ventricle; LVEF, LV ejection fraction; LVFS, LV fractional shortening; LVIDd, LV internal dimension in diastole; LVIDs, LV internal dimension in systole; LVM, left ventricular mass; LVPW, the thickness of LV posterior wall; LVSP, LV systolic pressure; SHRs, spontaneously hypertensive rats; TLR4, Toll like receptor 4; TNF-α, tumor necrosis factor-α; IL-6, interleukin-6; IL-10, interleukin-10.

In conclusion, an eight-week administration of choline slowed the progression of hypertension and ameliorated the cardiac, renal and vascular damage in SHRs. These protective effects may be correlated with the improvement of vagal activity and the inhibition of inflammatory responses in SHRs by choline (Fig. 6). Our findings not only broaden understanding of the actions of choline in ameliorating cardiovascular damage but also suggest the potential of choline in the prevention and treatment of hypertension. Choline may be a promising adjunct therapeutic approach for the alleviation of cardiovascular damage in hypertension. In addition, suppressing the inflammatory response and improving vagal modulation may be promising targets in the treatment of hypertension.

## Materials and Methods

### Animals and experimental protocols

All experimental procedures and methods were performed in accordance with the Guidelines on the Care and Use of Laboratory Animals (National Institutes of Health publication no. 85-23, revised 1996) and were approved by the Ethics Committee of Xi’an Jiaotong University. Seven-week-old male SHRs and age-matched male WKY rats were purchased from Vital River Co., Ltd. (Beijing, China). The animals were housed under standard 12 hour day/12 hour night cycles with free access to food and water and were acclimated for one week before the experiment. The animals were assigned to the following experimental groups: (1) WKY group (WKY, n = 8); (2) WKY with choline treatment (WKY+Choline, n = 8); (3) SHR group (SHR, n = 8); (4) SHR with choline treatment (SHR+Choline, n = 8). In groups (2) and (4), the rats were injected with choline chloride (8 mg/kg/day) intraperitoneally for eight weeks. In groups (1) and (3), normal saline was injected intraperitoneally.

### Non-invasive blood pressure measurement

Systolic blood pressure (SBP) was measured by tail cuff using the BP-6A system (Chengdu Technology & Market Co., LTD., Chengdu, China) at baseline (8 weeks of age) and then every 2 weeks until the end of the study period. Briefly, conscious rats were placed in an adapted restrainer and acclimated to the incubator chamber at 37 °C for 30 minutes before the measurement of blood pressure. The aim of the procedure was to calm the animals and dilate the tail blood vessels for obtaining stable and obvious waves of blood pressure. Then, the arterial blood pressure measurements were performed by using the manometer sleeve and pulse detector on the tail. For each rat, the blood pressure was measured once every 5 minutes, and the blood pressure measurements were carried out at least three times to successfully obtain valid values. For each rat, the mean values of the three successful measurements were used for further analysis.

### Echocardiography

Cardiac morphology and function were assessed by transthoracic two-dimensional echocardiography (Philips iE33, Philips, Bothell, USA) in rats anaesthetized with sodium pentobarbital (50 mg/kg, intraperitoneally) at the end of the experiment. The left ventricle ejection fraction (LVEF), LV fractional shortening (LVFS), LV internal dimension in systole and diastole (LVIDs and LVIDd), thickness of the LV posterior wall (LVPW), end-systolic volume (ESV), end-diastolic volume (EDV), and cardiac output (CO) were obtained.

### Haemodynamic parameters and baroreflex sensitivity analysis

All measurements were obtained in sodium pentobarbital-anaesthetized rats (50 mg/kg, intraperitoneally). After the induction of anaesthesia, the haemodynamic parameters were recorded and evaluated with a polygraph recorder (AD Instruments, Sydney, New South Wales, Australia). Briefly, a polyethylene catheter with one end connected to pressure transducers was inserted into the right carotid artery or the LV cavity to measure heart rate (HR), heart period (HP), mean arterial pressure (MAP), diastolic blood pressure (DBP), maximum rate of rise/descent of left ventricular pressure (±dp/dt_max_), LV systolic pressure (LVSP) and LV end-diastolic pressure (LVEDP), and another catheter was inserted into the left femoral vein for drug administration. After the haemodynamic parameters were recorded, a dose of phenylephrine (2~5 μg/kg, intravenously) was injected to raise the SBP of the rats between 20 and 40 mmHg. The relationship between HP and SBP was determined using a linear regression analysis. The slope of HP/SBP is used as an index for baroreflex sensitivity (BRS) (ms/mmHg)^66^.

### Collection of blood and tissue samples

After the BRS measurements, blood drawn from the abdominal aorta was collected and centrifuged at 3000 rpm for 20 minutes at 4 °C, and the supernatant serum was divided into aliquots and frozen at −80 °C until analysis.

The hearts, thoracic aortas and left kidneys were excised rapidly and washed with 4 °C phosphate-buffered saline (PBS) (137 mM NaCl, 2.7 mM KCl, 10 mM Na_2_HPO_4_, and 2 mM KH_2_PO_4_ at pH 7.4). The right ventricular free wall and atrial appendages were dissected away before weighing the left ventricle. After that, the remaining left ventricles were weighed and frozen in liquid nitrogen and stored at −80 °C.

### Mesenteric artery preparation and isometric tension measurement

The superior mesenteric artery was gently isolated and immersed immediately in cold oxygenated Krebs’ solution (119 mM NaCl, 1 mM MgCl_2_, 4.7 mM KCl, 1.2 mM KH_2_PO_4_, 2.5 mM CaCl_2_, 25 mM NaHCO_3_, and 11 mM D-glucose at pH 7.4). The adherent fat and tissue were carefully removed from the arteries, and the arteries were then cut into approximately 2–3 mm segments with a dissection microscope. The luminal surface was not damaged in the operation. Isometric tension was measured as previously described^66^,^67^. The arterial rings were immersed in organ chambers filled with Krebs’ solution (gassed with a mixture of 95% air and 5% CO_2_) maintained at 37 °C. Each ring was mounted on two L-shaped stainless steel holders. One holder was fixed on the organ bath, and the other was connected to a force displacement transducer (Beijing Aeromedicine Engineering Research Institute, Beijing, China) attached to a Taimeng BL-420F biotic signal collection and analysis system (Taimeng Instruments, Chengdu, China) to continuously record the isometric tension. The mounted superior mesenteric artery rings were equilibrated for 90 minutes. All the rings were stretched to a 0.5 g resting tension, an optimal tension that was determined previously^44^. The bath solution was replaced every 15 minutes in the resting period. At the end of the equilibration period, each ring was exposed to a high-K^+^ Krebs’ solution (prepared by substituting NaCl with an equimolar amount of KCl in the Krebs’ solution) for testing the contractile capacity. The rings were considered viable and were used only if high-K^+^ contractions were obtained twice reproducibly.

### Vascular reactivity studies

To investigate the effect of choline on vascular reactivity, we used the endothelium-intact mesenteric arterial rings as previously described^68^. Briefly, the mesenteric artery rings were used to generate concentration–response curves to 5-hydroxytryptamine (5-HT, 10^−8^–10^−4^ M) and phenylephrine (PE, 10^−8^–10^−4^ M). In addition, the endothelium-dependent and endothelium-independent relaxations were evaluated by measuring the responses to cumulative concentrations of either acetylcholine (ACh, 10^−10^–10^−5^ M) or sodium nitroprusside (SNP, 10^−10^–10^−5^ M) in mesenteric arteries pre-constricted with PE (10^−5^ M). After generation of each concentration–response curve, the rings were washed 3 times with fresh Krebs’ solution and then equilibrated for 45 minutes in Krebs’ solution before exposure to the next agent.

### Haematoxylin and eosin staining

Rat superior mesenteric arteries, thoracic aortas, hearts and kidneys were fixed in a 20% formaldehyde PBS solution for 24 hours. Then, the tissues were dehydrated, embedded in paraffin and then cut into 5-μm sections with a Leica RM-2135 microtome (Leica, Bensheim, Germany) for haematoxylin and eosin (H&E) staining. The heart section images were quantitatively analysed to determine cardiomyocyte area using Image-Pro Plus 6.0 (Media Cybernetics, Silver Spring, USA). To identify pathological changes of the renal tissues, the tissues were reviewed in a blinded manner and scored using a semiquantitative scoring system, as previously described^69^. We randomly selected 10 fields of the cortex and the outer stripe of the outer medulla. The renal damage was graded with an arbitrary score of 0–4 as follows: no injury (0); mild, 0–25% (1); moderate, 25–50% (2); severe, 50–75% (3); and very severe, 75–100% (4).

### Transmission electron microscopy

The superior mesenteric arteries were carefully isolated and fixed with 2.5% glutaraldehyde in 0.1 M phosphate buffer (pH 7.2–7.4) for 2 hours at 4 °C. Then, the arteries were postfixed with 1% osmium tetraoxide, dehydrated in a graded ethanol series and embedded in epoxy resin and then sliced into ultrathin sections (1 μm). After being counterstained with uranyl acetate and lead citrate, the sections were examined by a transmission electron microscope (H-7650; Hitachi, Tokyo, Japan).

### Measurement of serum ACh level

The serum concentration of ACh was determined by a commercially available kit (Jiancheng Bioengineering Institute, Nanjing, China), by following the manufacturer’s instructions. Absorbance was measured at 550 nm on a Stat Fax 2100 spectrophotometer (Awareness Technology, Palm City, FL).

### Western blot

Western blot analyses were performed to determine the protein expression of inflammatory factors in the mesenteric arteries according to the following protocols. The mesenteric artery tissue was homogenized in ice-cold RIPA buffer (Beyotime Biotech, Haimen, China) containing 1 mM phenylmethylsulfonyl fluoride. The protein concentrations were quantified using a Bicinchoninic acid protein assay kit (Beyotime Biotech). Equal amounts (30 μg/lane) of protein were loaded and separated by 10% SDS-PAGE and then electrotransferred to a polyvinylidene difluoride membrane. (Millipore, Billerica, MA, USA). Nonspecific binding to the membrane was blocked by incubating the membrane in Tris-buffered saline containing 5% non-fat milk and 0.1% Tween 20 at room temperature for 1 hour. TLR4 (100 kDa), IL-6 (23 kDa), IL-10 (19 kDa), TNF-α (17 kDa) and GAPDH (36 kDa) were detected by a rabbit polyclonal antibody against TLR4 (diluted 1:500; Bioworld Technology, St. Louis Park, MN, USA), a rabbit polyclonal antibody against IL-6 (diluted 1:500; Bioworld Technology), a rabbit polyclonal antibody against IL-10 (diluted 1:1000; Bioworld Technology), a rabbit polyclonal antibody against TNF-α (diluted 1:500; Bioworld Technology), and a mouse monoclonal antibody against GAPDH (diluted 1:5000, CMCTAG, Milwaukee, WI, USA), respectively. After incubation of the membrane with the appropriate horseradish peroxidase (HRP)-linked secondary antibody (diluted 1:5000; Signalway Antibody, College Park, MD, USA), immunoreactive bands were visualized by ECL-Plus reagent (Millipore) and then exposed to Biomax L film (Fuji, Tokyo, Japan). The bands were quantified by densitometry using Quantity One software (Bio-Rad Laboratories, Berkeley, CA, USA). The western blot analysis was repeated six independent times for each target protein in each group. Densitometric values were obtained and normalized to the average GAPDH value.

### Statistical analysis

The data are expressed as the mean ± SEM. A two-way ANOVA followed by Tukey’s post hoc test for multiple comparisons was used to determine the significance of differences, and *P* < 0.05 was defined to be statistically significant. Statistical analyses were performed with GraphPad software Prism 5 (GraphPad Software Inc, La Jolla, CA).

## Additional Information

**How to cite this article**: Liu, L. *et al*. Choline ameliorates cardiovascular damage by improving vagal activity and inhibiting the inflammatory response in spontaneously hypertensive rats. *Sci. Rep.* **7**, 42553; doi: 10.1038/srep42553 (2017).

**Publisher's note:** Springer Nature remains neutral with regard to jurisdictional claims in published maps and institutional affiliations.

## Supplementary Material

Supplementary Figure

## Figures and Tables

**Table 1:** Echocardiographic parameters in WKY rats and SHRs treated with choline or saline.

Variables	WKY (n = 8)	WKY+Choline (n = 8)	SHR (n = 8)	SHR+Choline (n = 8)
LVEF (%)	88.54 ± 2.04	87.74 ± 1.15^&^	77.30 ± 2.66^***^	83.18 ± 2.15^##^
LVFS (%)	53.14 ± 2.85	51.52 ± 1.91^&^	40.86 ± 2.41^***^	45.98 ± 2.46^#^
LVIDd (mm)	5.32 ± 0.38	5.57 ± 0.26	6.36 ± 0.14^***^	5.74 ± 0.22^##^
LVIDs (mm)	2.84 ± 0.29	2.86 ± 0.24	3.62 ± 0.33^***^	3.17 ± 0.38^#^
LVPW (mm)	1.38 ± 0.08	1.38 ± 0.09	1.55 ± 0.16^*^	1.36 ± 0.07^#^
EDV (ml)	0.41 ± 0.04	0.43 ± 0.05	0.57 ± 0.06^***^	0.48 ± 0.08^#^
ESV (ml)	0.05 ± 0.02	0.06 ± 0.01	0.12 ± 0.01^***^	0.08 ± 0.02^#^
CO (L/min)	0.12 ± 0.01	0.13 ± 0.01	0.18 ± 0.01^***^	0.14 ± 0.01^#^

CO, cardiac output; EDV, end diastolic volume; ESV, end-systolic volume; LV, left ventricle; LVEF, LV ejection fraction; LVFS, LV fractional shortening; LVIDd, LV internal dimension in diastole; LVIDs, LV internal dimension in systole; LVPW, LV posterior wall thickness. Values are expressed as mean ± SD.^*^P < 0.05, ^***^P < 0.001 versus WKY; ^#^*P* < 0.05, ^##^*P* < 0.01 versus SHR; & *P* < 0.05 versus SHR+Choline.
