# Supplementary material for: Choline ameliorates cardiovascular damage by improving vagal activity and inhibiting the inflammatory response in spontaneously hypertensive rats
Source: Sci Rep. 2017 Feb 22;7:42553. doi: 10.1038/srep42553 (PMC5320519; doi:10.1038/srep42553)

**Choline ameliorates cardiovascular damage by improving vagal activity and** **inhibiting the inflammatory response in spontaneously hypertensive rats**

Longzhu Liu, Yi Lu, Xueyuan Bi, Man Xu, Xiaojiang Yu, Runqing Xue, Xi He*, Weijin Zang*

Department of Pharmacology, School of Basic Medical Sciences, Xi’an Jiaotong University Health Science Center, Xi’an, Shaanxi, 710061, People’s Republic of China.

**Corresponding Author**:

***PhD. Xi He**. P.O. Box 77^#^, No.76 Yanta West Road, Department of Pharmacology, School of Basic Medical Sciences, Xi’an Jiaotong University Health Science Center, Xi’an, Shaanxi, 710061, People’s Republic of China.

***Prof. Weijin Zang**. P.O. Box 77^#^, No.76 Yanta West Road, Department of Pharmacology, School of Basic Medical Sciences, Xi’an Jiaotong University Health Science Center, Xi’an, Shaanxi, 710061, People’s Republic of China.

Tel: +86-29-82655150; E-mail: zwj@mail.xjtu.edu.cn

**Supplementary Figure**


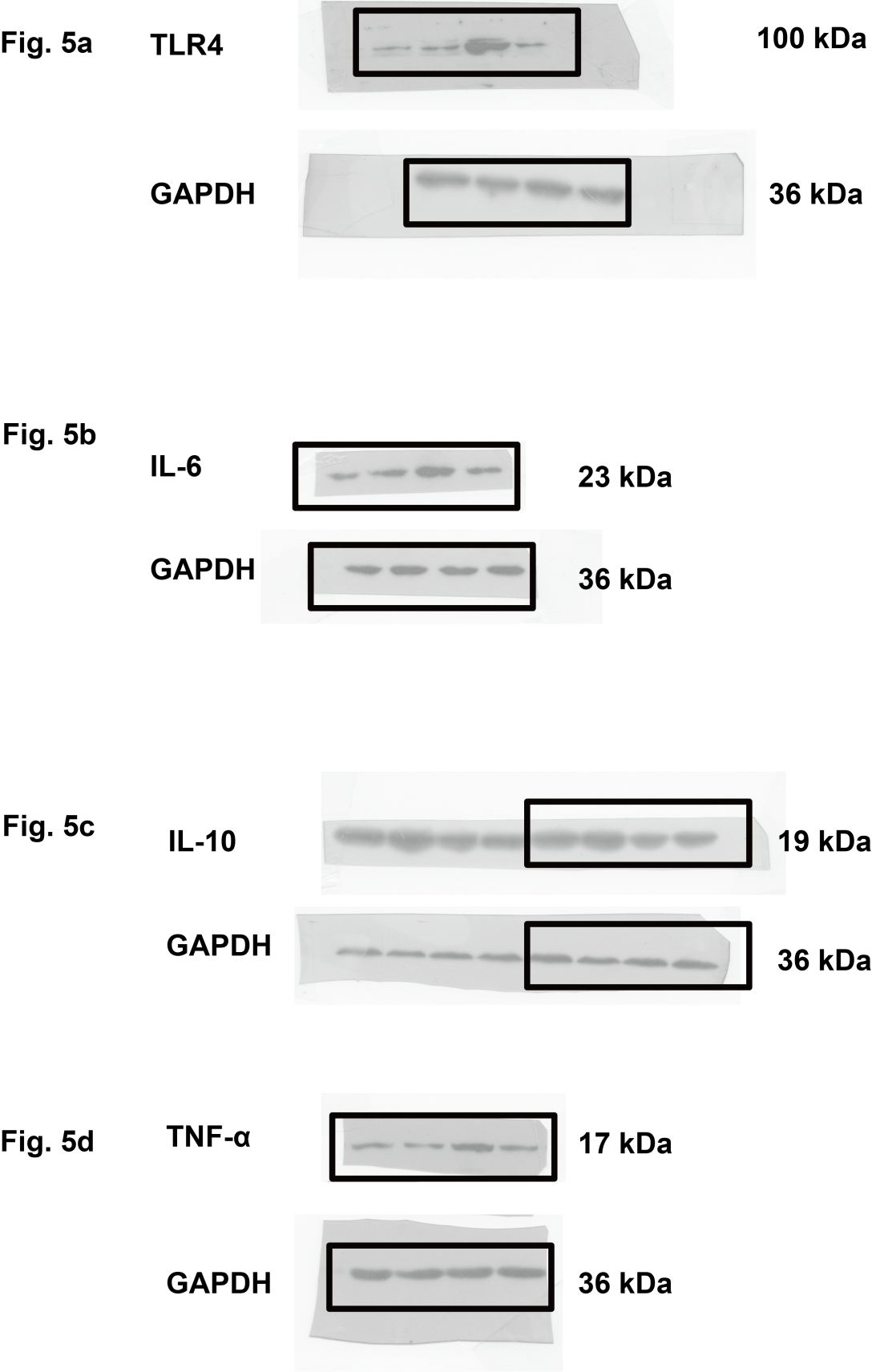

Supplement: Supplementary Figure [file srep42553-s1.doc]
